# Association of ethylene oxide exposure and obstructive sleep apnea

**DOI:** 10.1265/ehpm.24-00248

**Published:** 2025-02-06

**Authors:** Shanni Ma, Shangfen Xie

**Affiliations:** 1 Department of Respiratory and Critical Care Medicine, The First Affiliated Hospital of Ningbo University, Zhejiang 315010, China; 2 Department of Hepatopancreatobiliary Surgery, The First Affiliated Hospital of Ningbo University, Zhejiang 315010, China

**Keywords:** Ethylene oxide, Obstructive sleep apnea, NHANES, Epidemiology

## Abstract

**Background:**

Ethylene oxide (EO) is a widely utilized industrial compound known to pose health hazards. Although its carcinogenic characteristics have been thoroughly investigated, recent findings indicate possible links to respiratory disease. The correlation between EO exposure and the likelihood of developing obstructive sleep apnea (OSA) in individuals remains unclear. The study aimed to explore the association between EO exposure and OSA within the broader US population.

**Methods:**

From 2015 to 2020, 4355 participants were analyzed cross-sectionally in the National Health and Nutrition Examination Survey (NHANES). As the primary indicator of EO exposure, hemoglobin adducts of EO (HbEO) were used in this study. The relationship between EO exposure and OSA prevalence was assessed using weighted multivariable regression analysis and smoothing curve fitting. Using subgroup analysis and interaction tests, we investigated whether this association remained consistent across populations.

**Results:**

According to the study, higher HbEO level was positively correlated with a higher prevalence of OSA. Compared to the first HbEO quartile (Q1), participants within the highest quartile (Q4) presented a higher OSA prevalence in the fully model (OR = 1.32, 95% CI: 1.08–1.62, P = 0.01, P for trend = 0.001). This correlation was particularly evident among females and individuals who are insufficiently physically active.

**Conclusions:**

This research found a positive relationship between the extent of exposure to EO and OSA prevalence among a representative sample of Americans.

## Introduction

Obstructive Sleep Apnea is the disease characterized by recurring occurs of the upper airway collapsing and becoming obstructed during sleep, causing sleep interruptions, awakenings, sometimes a decrease in oxygen levels [1]. It is a common disease affecting an estimated one billion people worldwide [2]. An estimated 425 million individuals are thought to suffer from moderate to severe OSA, a condition typically advised for intervention [2]. The patients with OSA are characterized by loud snoring, gasping for air, frequent interruptions in sleep, and excessive daytime fatigue, seriously affecting the lives of patients and partners [3]. Moreover, there is increasing evidence that OSA is linked to a range of disorders, including cardiovascular disorders, neurocognitive sequelae and mood disorders, seriously threatening people’s physical and mental health [4–8]. These intricate psychosomatic conditions carry substantial economic and social implications, suggesting they are poised to become an increasingly prominent global issue in the future. It is crucial to investigate and prevent the occurrence of OSA.

Ethylene oxide (EO) is an organic compound with global production and extensive utilization as a chemical precursor in the production of various products including antifreeze, detergents, glues, fabrics, solvents, and insecticides [9]. It is also frequently used for sterilizing medical devices and fumigating food items (like spices and nuts) and cosmetics [10]. EO reacts with hemoglobin to form HbEO, which is more stable and sensitive than the former and can be used as a blood biomarker to assess EO exposure [11, 12]. Despite its extensive use, there are significant concerns regarding the health hazards linked to EO. Research has demonstrated the carcinogenic properties of EO in laboratory and animal studies [13, 14]. Prolonged exposure to EO, particularly in industrial settings, has been associated with an increased risk of tumors in the hematopoietic system and breast cancer [15, 16]. Additionally, EO has alkylating properties and is thought to trigger genetic mutations and chromosomal abnormalities through the creation of adducts with DNA [17, 18]. Recent studies have indicated potential connections between EO exposure and a variety of health risks, such as diabetes [19], cardiovascular diseases [20], chronic obstructive pulmonary disease (COPD) [21], and metabolic syndrome [22], but more research is needed to explore these associations further. It is important to note that exposure to air pollution early in life, especially during pregnancy, may have adverse effects on the respiratory system of offspring [23, 24].

The connection between exposure to EO and the likelihood of OSA is not well understood. There is growing evidence suggesting that EO can trigger inflammatory reactions in various diseases, such as cardiovascular disease and lipid metabolism disorders [20, 25, 26]. Given the significant role of inflammation in OSA, it is speculated that EO could play a part in the development of OSA. Therefore, the study utilizes data from the National Health and Nutrition Examination Survey (NHANES) to investigate the link between EO exposure and OSA.

## Methods

### Study population

**Fig. 1:**
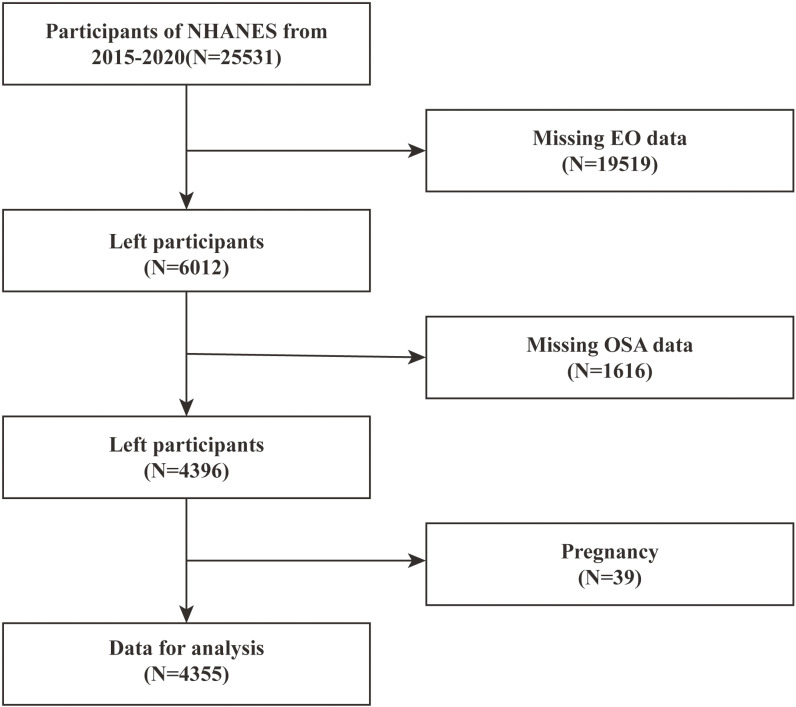
Flowchart of participants selection. NHANES, National Health and Nutrition Examination Survey

The NHANES survey, a nationally representative study overseen by the Centers for Disease Control and Prevention, has been approved by the Research Ethics Review Board of the National Center for Health Statistics (NCHS) for implementation. It involves interviews, physical exams, lab testing, and health-related questionnaires with oversampling techniques. The data is accessible on the NHANES website. All participants provided written consent. We included a total of 25531 participants from three NHANES cycles (2015–2016 and 2017–2020). After combining the databases, we removed individuals with incomplete information on EO exposure (n = 19619), missing OSA data (n = 1616), and pregnant females (n = 39). This left us with 4355 participants for the subsequent analyses (Fig. 1).

### Assessment of OSA

Based on previous research, a diagnosis of obstructive sleep apnea (OSA) is made if an individual responds affirmatively to any of the three NHANES questions: (1) feeling excessive tiredness during the daytime despite getting a minimum of 7 hours of sleep each night, reported sixteen to thirty times; (2) having instances of gasping, snorting, or breath cessation at least 3 times a week; (3) snoring at least three times per week.

### Measurement of EO

EO exposure is measured using HbEO, which has a longer biological half-life. HbEO levels were measured using the NHANES Laboratory/Medical Technologist Procedures Manual. Before examination at the National Center for Environmental Health, participants’ wash-packed erythrocyte specimens were stored at −30 °C. Second, HbEO was measured using the modified Edman technique and high-performance liquid chromatography with tandem mass spectrometry. Finally, HbEO was measured in pmol adducts per gram Hb.

### Covariates

Covariates included gender, age, ethnicity, education status, poverty income ratio (PIR), marital status, alcohol intake, physical activity, smoking status, hypertension, diabetes, asthma, cancer, and body mass index (BMI). A comprehensive account of the measurement processes was revealed on www.cdc.gov/nchs/nhanes.

### Statistical analysis

Based on CDC guidelines, statistical analyses in this study were conducted incorporating appropriate NHANES sampling weights. The mean and standard deviation for continuous variables were reported, and the percentages for categorical variables. We used a weighted t-test or a weighted chi-square test to assess the difference. As HbEO levels were not distributed normally, the data were normalized via natural logarithm transformation, which was then categorized into quartiles. Three different models of the connection between ln-transformed HbEO levels and OSA were investigated using multivariate logistic regression models. Model 1 did not account for covariates, while Model 2 was adjusted for race, gender, and age. Model 3 was adjusted for gender, age, ethnicity, education status, PIR, marital status, drinking status, physical activity, smoking status, hypertension, diabetes, asthma, cancer, and BMI. The nonlinear connection between ln-transformed HbEO levels and OSA was further analyzed using smooth curve fitting. An analysis of subgroups stratified based on gender, age, race, diabetes, hypertension, smoking status, physical activity, and alcohol intake was also assessed using stratified multivariate regression. Statistical significance was determined by P < 0.05. Empower software (www.empowerstats.com) and R version (www.Rproject.org) were conducted for all analyses.

## Results

### Baseline characteristics

Table 1 displays the enrollment of 4355 participants in the current research sourced from the NHANES database spanning the years 2015 to 2020. The prevalence of OSA was found to be 50.47%, with 2198 individuals diagnosed with OSA and 2157 without. The participants were categorized into four groups according to the quartiles of ln-transformed HbEO values: Q1 group (<2.78, n = 1078), Q2 group (2.78–3.08, n = 1096), Q3 group (3.08–3.69, n = 1092), and Q4 group (≥3.69, n = 1089). The data in Table 1 indicates significant variations in age, sex, ethnicity, education status, marital status, PIR, alcohol consumption, BMI, hypertension, diabetes, asthma, cancer history, OSA diagnosis, and smoking status across the 4 groups (P < 0.001). But no notable significance was found in terms of physical activity (P > 0.05).

**Table 1:** Baseline characteristics of participants according to quartiles of ln-transformed HbEO.

**Variables**	**Quartile 1** **(<2.78)**	**Quartile 2** **(2.78–3.08)**	**Quartile 3** **(3.08–3.69)**	**Quartile 4** **(>3.69)**	**P**
N	1078	1096	1092	1089	
Age (year)	47.16 ± 19.37	48.03 ± 20.59	47.92 ± 19.18	45.40 ± 16.93	0.006
PIR	2.80 ± 1.64	2.64 ± 1.60	2.68 ± 1.64	2.03 ± 1.49	<0.001
BMI	30.09 ± 7.46	29.71 ± 7.29	29.09 ± 6.91	28.40 ± 7.12	<0.001
Gender, n (%)					<0.001
Male	496 (46.01%)	533 (48.63%)	505 (46.25%)	665 (61.07%)	
Female	582 (53.99%)	563 (51.37%)	587 (53.75%)	424 (38.93%)	
Cancers, n (%)					0.032
No	942 (87.38%)	968 (88.32%)	987 (90.38%)	988 (90.73%)	
Yes	136 (12.62%)	128 (11.68%)	105 (9.62%)	101 (9.27%)	
Race, n (%)					<0.001
Mexican American	158 (14.66%)	205 (18.70%)	167 (15.29%)	96 (8.82%)	
White	601 (55.75%)	522 (47.63%)	382 (34.98%)	429 (39.39%)	
Black	186 (17.25%)	204 (18.61%)	267 (24.45%)	384 (35.26%)	
Other Races	133 (12.34%)	165 (15.05%)	276 (25.27%)	180 (16.53%)	
Education level, n (%)					<0.001
Less than high school	122 (11.32%)	140 (12.77%)	129 (11.81%)	88 (8.08%)	
High school	325 (30.15%)	363 (33.12%)	377 (34.52%)	524 (48.12%)	
Above high school	631 (58.53%)	593 (54.11%)	586 (53.66%)	477 (43.80%)	
Asthma, n (%)					<0.001
Yes	155 (14.38%)	137 (12.50%)	154 (14.10%)	214 (19.65%)	
No	923 (85.62%)	959 (87.50%)	938 (85.90%)	875 (80.35%)	
Smoking status, n (%)					<0.001
Never	788 (73.10%)	808 (73.72%)	796 (72.89%)	244 (22.41%)	
Current	290 (26.90%)	288 (26.28%)	296 (27.11%)	845 (77.59%)	
Diabetes, n (%)					0.002
No	960 (89.05%)	934 (85.22%)	913 (83.61%)	947 (86.96%)	
Yes	118 (10.95%)	162 (14.78%)	179 (16.39%)	142 (13.04%)	
Hypertension, n (%)					0.002
No	728 (67.53%)	748 (68.25%)	727 (66.58%)	667 (61.25%)	
Yes	350 (32.47%)	348 (31.75%)	365 (33.42%)	422 (38.75%)	
Physical Activity, n (%)					0.282
Never	303 (28.11%)	311 (28.38%)	348 (31.87%)	306 (28.10%)	
Moderate	284 (26.35%)	294 (26.82%)	290 (26.56%)	278 (25.53%)	
Vigorous	491 (45.55%)	491 (44.80%)	454 (41.58%)	505 (46.37%)	
Alcohol, n (%)					<0.001
No	688 (63.82%)	777 (70.89%)	830 (76.01%)	657 (60.33%)	
Yes	390 (36.18%)	319 (29.11%)	262 (23.99%)	432 (39.67%)	
Marital Status, n (%)					<0.001
Cohabitation	717 (66.51%)	708 (64.60%)	699 (64.01%)	587 (53.90%)	
Solitude	361 (33.49%)	388 (35.40%)	393 (35.99%)	502 (46.10%)	
OSA, n (%)					0.011
No	531 (49.26%)	555 (50.64%)	574 (52.56%)	497 (45.64%)	
Yes	547 (50.74%)	541 (49.36%)	518 (47.44%)	592 (54.36%)	

PIR: Poverty income ratio

Data of continuous variables are shown as mean (95% CI); P-value was calculated by linear regression.

Data of categorical variables are shown as percentage (95% CI), P-value was calculated by Chi-square test.

### Association of EO and OSA

**Fig. 2:**
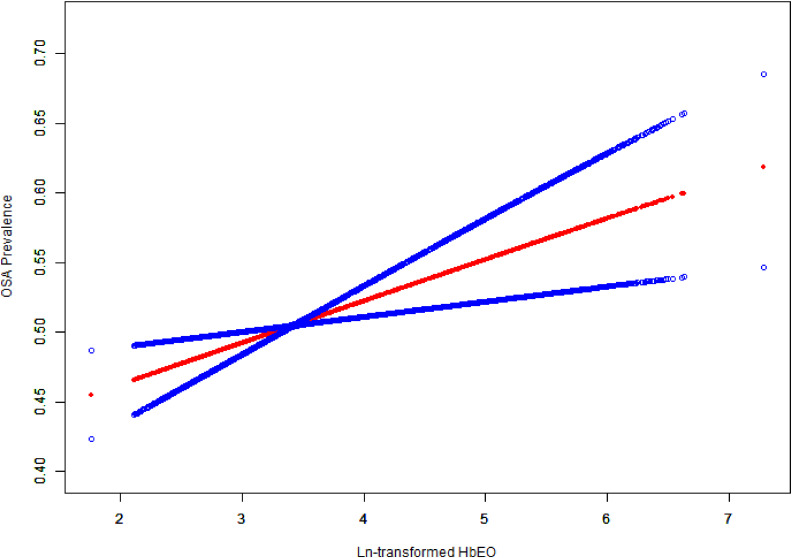
Smoothing curve fitting between the ln-transformed HbEO and OSA prevalence. Adjusted for all covariates include gender, age, ethnicity, education status, poverty income ratio (PIR), marital status, alcohol intake, physical activity, smoking status, hypertension, diabetes, asthma, cancer, and body mass index (BMI).

Our results indicated a significant positive relationship between ln-transformed HbEO and OSA prevalence (Table 2). Fully adjusted (Model 3) showed a stable positive association (OR = 1.13; 95% CI: 1.05–1.22; p < 0.01), implying that there was a 13% increase in OSA prevalence for each unit increase in ln-transformed HbEO. Additionally, ln-transformed HbEO values were changed to categorical variables based on quartiles. In the fully adjusted model, individuals in the fourth quartile had a higher prevalence of OSA compared to the first quartile (OR = 1.32, 95% CI: 1.08–1.62, P = 0.01, P for trend < 0.01). An analysis of the nonlinear connection between ln-transformed HbEO and OSA was investigated using weighted multivariable regression analysis and smooth curve fittings. The results demonstrated a positive correlation between ln-transformed HbEO and OSA prevalence (Fig. 2).

**Table 2:** Association between ln-transformed HbEO and OSA prevalence

**OSA**	**Model 1**		**Model 2**		**Model 3**	
	OR (95%CI)	P	OR (95%CI)	P	OR (95%CI)	P
Ln-HbEO	1.07 (1.01, 1.14)	0.03	1.09 (1.02, 1.16)	0.01	1.13 (1.05, 1.22)	0.002
Categories						
Q1	1		1		1	
Q2	0.95 (0.80, 1.12)	0.52	0.92 (0.77, 1.09)	0.34	0.96 (0.80, 1.15)	0.64
Q3	0.88 (0.74, 1.04)	0.12	0.88 (0.74, 1.05)	0.17	0.94 (0.78, 1.13)	0.49
Q4	1.16 (0.98, 1.37)	0.09	1.20 (1.01, 1.43)	0.04	1.32 (1.08, 1.62)	0.01
P for trend	0.01		0.003		0.001	

The Ln-HbEO was converted from a continuous variable into a categorical variable (quartiles).

Model 1 = no covariates were adjusted.

Model 2 = Model 1 + age, gender, and race were adjusted.

Model 3 = Model 2 + education level, PIR, marital status, alcohol consumption, physical activity, smoking status, hypertension, diabetes, asthma, cancer, BMI.

### Subgroup analysis

**Fig. 3:**
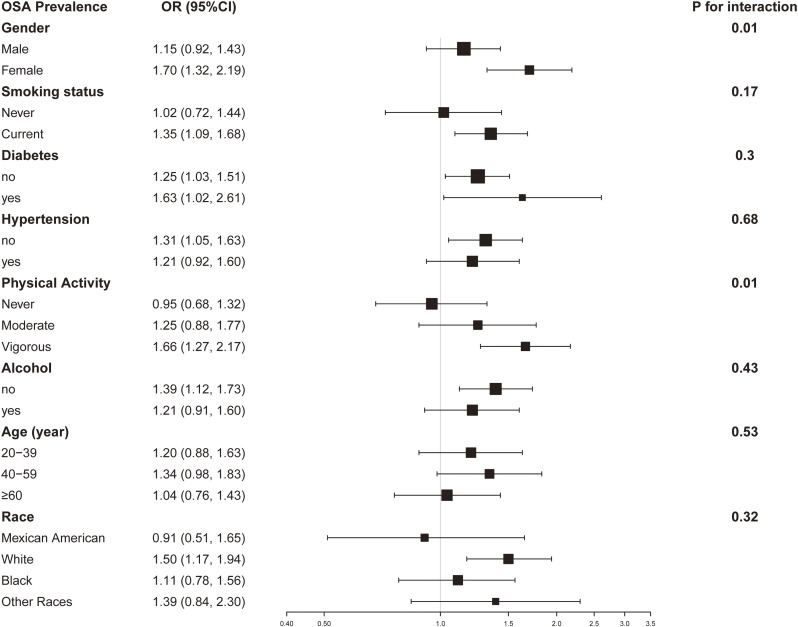
Subgroup analysis for the association between ln-transformed HbEO and OSA. Models were adjusted for age (years), sex, BMI (<25 or ≥25 kg/m^2^), race/ethnicity (Mexican American, White, Black, Other Race), educational level (less than high school, high school, above high school), marital status, smoking status (current or never), drinking status (yes, or no), PIR, diabetes (yes or no), hypertension (yes or no), asthma, cancer, and physical activity (vigorous, moderate, or never).

Subgroup analysis and interaction test were conducted to determine whether the association between ln-transformed HbEO and OSA is stable and what variations may exist across different populations. As shown in Fig. 3, the relationship between ln-transformed HbEO and OSA differed significantly by sex subgroup and physical activity subgroup (P for interaction < 0.01). And observed interaction did not demonstrate statistical significance following stratification based on age, ethnicity, alcohol drinking, smoking situation, diabetes mellitus, and hypertension (P for interaction > 0.05).

## Discussion

In this research, we examined information from the NHANES data base, which represents the American population between 2015 and 2020. To our knowledge, this study represents the initial attempt to explore the association between the extent of exposure to EO and the prevalence of OSA. The results showed that individuals diagnosed with OSA are exposed to higher concentrations of EO in comparison with those without OSA. Additionally, we found a significant positive correlation between in vivo HbEO value and OSA prevalence. Upon adjusting for pertinent variables including age, sex, race, alcohol consumption, marital status, educational background, smoking status, annual family income, and BMI, our analysis indicated a positive relationship between HbEO levels and OSA prevalence. Notably, individuals in the higher quartiles of HbEO levels demonstrated a higher prevalence of OSA in comparison to those in the lowest quartile. The subgroup analysis of our research indicated inconsistent associations between EO and OSA across gender and exercise levels.

EO is a reactive epoxide commonly utilized for sterilization purposes in various industries, including food, seasonings, and medical instruments. And it is used to produce chemicals such as glycols, ethoxylates, and acrylonitrile [27]. Workers in specific occupations, particularly those involved in sterilizing medical equipment, are in danger of occupational exposure to higher concentrations of EO [11, 28]. Additionally, EO is present in cigarette smoke, posing the potential risk of exposure to general people through volatile organic chemicals and secondhand smoke [29, 30]. Exposure to EO has been linked to various health risks, with the International Agency for Research on Cancer and the US Environmental Protection Agency categorizing it as a cancer-causing agent in 2016 [31]. Studies have shown that EO exposure is carcinogenic in animals and has been linked to malignant disorders in humans, including lymphoid leukemia [32] and breast cancer [33]. While existing research has primarily focused on occupational exposures to EO, there is limited investigation into potential health risks related to EO exposure in the general population. There are various pathways through which individuals may come into contact with ethylene oxide. One such route is the endogenous production of ethylene oxide within the body, which occurs through processes like bacterial synthesis by the gastrointestinal pathway or generated by the liver. Consequently, everybody experiences some level of exposure to EO, irrespective of external sources of ethylene oxide in the environment [34, 35]. In individuals who do not smoke, internal EO exposure is the primary contributor to overall exposure levels. Exogenous exposure to ethylene oxide also has an indirect effect on endogenous production pathways, potentially through an oxidative stress mechanism, and the latter pathway may be more important than the former in terms of overall exposure [27]. However, more research is needed to distinguish the effects of exogenous and endogenous EO exposure on OSA. Several components of metabolic syndrome have been proven to be associated with EO [19, 36, 37], and EO has also been proven to be a risk factor for metabolic syndrome. OSA is highly correlated with metabolic syndrome [38–40], so there is reason to believe that OSA and EO are closely related. In this research involving a representative sample of the American population, the connection between EO exposure and OSA prevalence was examined for the first time in the general populace. The findings of this investigation revealed a significant correlation between higher EO exposure and a higher prevalence of OSA compared to lower exposure levels. These results can offer valuable insights for future study endeavors.

The precise biological mechanism linking exposure to EO and OSA is exactly uncertain. Various research has highlighted a notable correlation between EO and inflammation. Research has shown that exposure to EO in rodents can lead to inflammatory lesions in various organs. In 1984, Lynch first reported that chronic inhalation of EO caused inflammatory lesions in multiple organs of F344 rats [14]. And exposure to EO can promote the occurrence of pulmonary fibrosis in rodents [41]. Zeng and colleagues have suggested that exposure to EO may change fatty acid metabolism and cause inflammatory reactions, which may lead to the occurrence of cardiovascular disease [20]. Furthermore, EO exposure has been linked to changes in serum lipid profiles across systemic inflammatory responses [26]. Previous animal studies have indicated that EO exposure can reduce intracellular glutathione levels and increase hepatic lipid peroxidation. These changes are well-established indicators of oxidative stress in live organisms [42–44]. The latest research reports that EO affects the formation of kidney stones through inflammation, and physical activity may alter this connection somewhat [45], and this is consistent with the results of our subgroup analysis. Inflammatory response and oxidative stress are fundamental underlying mechanisms in OSA pathology [46, 47]. In a limited clinical study with human participants, individuals diagnosed with OSA demonstrated disrupted triglyceride metabolism [48]. Apart from influencing levels of cholesterol in the bloodstream, OSA could potentially impact lipid metabolism by promoting the oxidatively damaged lipids’ synthesis [49]. The severity of OSA appeared to escalate in correlation with the advancement of menopause in a dose-dependent manner [50]. This discrepancy between genders tends to diminish post-menopause, indicating a potential influence of menopausal status on the characteristics of OSA. The involvement of sex hormones is hypothesized to be a significant factor in these observations [51]. This may explain the change in the correlation between EO and OSA by sex. In conclusion, the relationship between EO and OSA may be mediated by oxidative stress and inflammation. However, further research is still needed to determine the underlying biological mechanism linking EO exposure to OSA.

This study represents the inaugural investigation utilizing the NHANES database to evaluate the correlation between levels of EO exposure and OSA within a nationally representative sample of the United States populace. Rigorous measurement techniques and standardized quality assessments were applied to all data within the database. This research contributes novel insights to the understanding of OSA pathogenesis. Nevertheless, the study is subject to certain limitations. Primarily, being a retrospective analysis reliant on the NHANES database, inherent selection biases may be present. Furthermore, due to the cross-sectional nature, establishing a comprehensive causal connection between EO and OSA risk is challenging, necessitating further prospective investigations. Moreover, dependence on self-reported data from NHANES participants introduces potential recall biases. Although the NHANES survey has a classification of work, it is not possible to tell whether it is an individual who has been exposed to EO, which is another limitation. Lastly, the study lacked precise information regarding the timing of OSA onset and the specific OSA subtypes, highlighting the need for future research to differentiate between OSA types associated with EO exposure.

## Conclusion

In conclusion, our findings indicate that higher levels of EO exposure were related to a higher prevalence of OSA, and this connection was more significant among females and adults who are insufficiently physically active. However, further investigation is needed to shed light on the causality of this connection.

## Abbreviations

EO: Ethylene oxide
OSA: Obstructive sleep apnea
SD: Standard deviations
COPD: Chronic obstructive pulmonary disease
CDC: Centers for Disease Control and Prevention
NHANES: National Health and Nutrition Examination Survey
HbEO: Hemoglobin adducts of EO
PIR: Poverty income ratio
BMI: Body mass index
NCHS: National Center for Health Statistics

## Declarations

### Availability of data and materials

The survey data are publicly available on the internet for data users and researchers throughout the world (www.cdc.gov/nchs/nhanes/).
